# 液相色谱-四极杆-飞行时间质谱法快速筛查与确证紫甘蓝中415种农药残留

**DOI:** 10.3724/SP.J.1123.2020.05006

**Published:** 2021-03-08

**Authors:** Yujie XIE, Hui CHEN, Lijuan GE, Siyu HUO, Chunlin FAN, Meiling Lü

**Affiliations:** 1.中国检验检疫科学研究院, 北京 100176; 1. Chinese Academy of Inspection and Quarantine, Beijing 100176, China; 2.北京合众恒星检测科技有限公司, 北京 100176; 2. Beijing Usi-Star Inspection Technology Co., Ltd., Beijing 100176, China; 3.安捷伦科技(中国)有限公司, 北京 100102; 3. Agilent Technologies (China) Limited, Beijing 100102, China

**Keywords:** 液相色谱-四极杆-飞行时间质谱, 筛查与确证, 欧盟能力验证, 农药残留, 紫甘蓝, liquid chromatography-quadrupole-time of flight-mass spectrometry (LC-QTOF/MS), screening and confirmation, European proficiency test, pesticide residues, red cabbage

## Abstract

应用液相色谱-四极杆-飞行时间质谱(LC-QTOF/MS)建立了一次进样可同时对紫甘蓝中415种农药残留进行快速筛查和准确确证的分析方法。实验采用1%(v/v)醋酸乙腈溶液提取,无水硫酸镁和氯化钠进行盐析,ZORBAX SB-C18色谱柱(100 mm×2.1 mm, 3.5 μm)分离,以0.1%(v/v)甲酸水溶液(含5 mmol/L乙酸铵)-乙腈为二元流动相进行梯度洗脱,应用LC-QTOF/MS在电喷雾电离、全离子MS/MS(All Ions MS/MS)扫描正模式下进行检测,基质匹配外标法定量分析。通过优化全自动MS/MS采集模式(Auto MS/MS)和全离子MS/MS采集模式下的不同参数,得到每种采集模式下的最佳条件。然后在2种不同采集模式的最佳条件下对比,最终选取All Ions MS/MS采集模式。实验结果表明,采用所建立的分析方法可以准确定性和定量筛查紫甘蓝中415种农药残留,所有415种农药在各自的范围内线性相关系数(*r*^2^)均大于0.990,其中411种农药的筛查限(SDL)≤5 μg/kg, 413种农药的定量限(LOQ)≤10 μg/kg。在1倍、2倍和10倍LOQ添加水平下,农药的回收率分别为65.7%~118.4%、72.0%~118.8%和70.2%~111.2%,相对标准偏差分别为0.9%~19.7%、0.2%~19.9%和0.6%~19.9%。将该方法应用于2019年欧盟能力验证项目的紫甘蓝样品中未知农药残留筛查方法和定量方法考核样的检测,所有添加农药均被准确定性筛查和定量检测,没有假阳性和假阴性。结果表明,该方法快速、准确、可靠,适用于对紫甘蓝中多种农药残留的高通量定性筛查和准确定量,可以扩展到其他果蔬基质中多农残的高通量筛查。

## Abstract (en)

An analytical method for the simultaneous rapid screening and accurate confirmation of 415 pesticide residues in red cabbages was established using liquid chromatography-quadrupole-time of flight-mass spectrometry (LC-QTOF/MS) with single acquisition. In the established method, the pesticides in red cabbage were extracted using acetonitrile-acetic acid (99∶1, v/v) and salted-out using anhydrous magnesium sulfate and sodium chloride. The resultant solution was then cleaned-up by automatic solid phase extraction using a Carbon/NH_2_ cartridge. The SPE cartridge was activated with 4 mL acetonitrile-toluene (3∶1, v/v) and the effluents were discarded. The resultant solution was transferred to the Carbon/NH_2_ cartridge, using 3×2 mL acetonitrile-toluene (3∶1, v/v) to wash the test sample concentrate bottle, and waited until the surface of the test sample concentrate liquid reached the top layer of anhydrous Na_2_SO_4_ before transferring the washing liquid to the cartridge. A 30-mL reservoir was attached to the upper part of the SPE cartridge and 25 mL acetonitrile-toluene (3∶1, v/v) was used to wash the SPE cartridge again. The eluent was evaporated in the glass tube in a water bath at 37 ℃ and shaking speed 150 r/min to reduce the volume to 0.5 mL. Nitrogen was used to dry the concentrates, and the residues were dissolved in 1.0 mL acetonitrile-water (3∶2, v/v), homogenized by ultrasonication, and passed through 0.22-μm filtering membrane before determination. The dissolved sample solution was loaded onto a ZORBAX SB-C18 column (100 mm×2.1 mm, 3.5 μm) and separated under gradient elution using 0.1% (v/v) formic acid aqueous solution containing 5 mmol/L ammonium acetate and acetonitrile as the binary mobile phase. The eluent from the column was further detected by QTOF/MS under electrospray positive ionization in the MS/MS scanning mode. A matrix-matched external calibration method was used for quantitation. By optimizing the different parameters under Auto MS/MS and All Ions MS/MS acquisition modes, the optimal conditions for All Ions MS/MS under each acquisition mode were obtained, which were then compared for selection of a better mode. The results demonstrated that the developed method can be used to accurately screen and quantify all 415 pesticides in red cabbage. The linear regression correlation coefficients (*r*^2^) for the 415 pesticides were all greater than 0.990 in the corresponding linear concentration range. In addition, the screening detection limits (SDL) of 411 pesticides were no more than 5 μg/kg, and the limits of quantification (LOQs) of 413 pesticides were no more than 10 μg/kg. At the spiked levels of LOQ, two-fold LOQ, and 10-fold LOQ, the recoveries were in the ranges of 65.7%-118.4%, 72.0%-118.8% and 70.2%-111.2%, with relative standard deviations (RSDs) in the ranges of 0.9%-19.7%, 0.2%-19.9% and 0.6%-19.9%, respectively. The method was applied to detect pesticide residues in the red cabbage samples provided by the 2019 European proficiency test project for unknown pesticide screening (EUPT-SM-11) and accurate quantitation (EUPT-FV-21). For EUPT-SM-11, all the spiked and incurred pesticides in red cabbage were qualified accurately, without false positives or false negatives. This is completely consistent with the final results published by the EU official. For EUPT-FV-21, there were 19 non-volatile pesticides that can be detected by LC-MS, which were then accurately quantitated with the corresponding pesticide standard. The results demonstrate that the proposed method is accurate and reliable. It is also rapid and time-saving, and can be used for high-throughput screening and quantitative determination of pesticide residues in cabbage. It can also be extended to other fruits and vegetable matrices.

农药残留是食品安全领域长期关注的问题之一。传统的农药残留主流检测技术有气相色谱-质谱法^[1]^、气相色谱-三重四极杆质谱法^[2,3,4,5,6,7]^和液相色谱-三重四极杆质谱法等^[8,9,10,11,12,13,14]^。随着农药种类的不断增加,目前世界常用农药已经增加到1100多种^[15]^,传统农药残留检测技术已经不能满足实际检测需求。

高分辨质谱以其精确质量数、高分辨率和不依赖标准品进行定性的优势^[16,17]^,在食品中农药残留高通量筛查方法中得到了广泛应用^[18,19]^。例如,赵志远等^[20]^应用液相色谱-四极杆-飞行时间质谱(LC-QTOF/MS)建立281种农药的一级精确质量数据库和二级谱图库,并采用该数据库和谱图库检索的方式,实现了苹果、番茄和甘蓝中281种农药残留的快速筛查。宋伟等^[21]^应用LC-QTOF/MS建立了204种农药的一级精确质量数据库和二级谱图库,通过化合物的精确质量数、保留时间、同位素比值等信息对检测结果进行自动检索,从而在无对照标准品的情况下实现了204种农药的定性鉴定。在这些方法中,通常需要2次进样筛查,即第一次进样后通过加合离子、保留时间等信息对化合物进行初步筛查,第二次则是针对初筛化合物建立二级采集方法,通过与二级谱图库比对进行化合物确证。尽管这种方式进行农残筛查可以获得较高的灵敏度,但是从快速、高效和节约成本的角度考虑,建立一次进样的筛查方法可以显著提高筛查效率^[22]^。

全自动MS/MS采集模式(Auto MS/MS)和全离子MS/MS采集模式(All Ions MS/MS)是LC-QTOF/MS中只需要一次进样便可对化合物进行筛查的两种采集模式。在Auto MS/MS模式中,输入加合离子信息和碰撞能后,质谱在一个循环时间内交替对一级质谱和二级质谱进行扫描,根据一级和二级质谱匹配情况对化合物进行确证^[23]^。在All Ions MS/MS采集模式中,选择多个碰撞能通道进行采集,通过分析各通道化合物共流出的特征,得到加合离子和碎片离子的共流出关系,根据目标物的保留时间、加合离子和碎片离子的精确分子量等信息与数据库进行检索比对,最终对化合物进行确证^[24,25]^。

紫甘蓝是一种常见的蔬菜,常见于蔬菜沙拉等非加热类菜品中,在其种植过程中通常会施用多种农药。研究表明,紫甘蓝属于碱性蔬菜,其紫红色叶片中色素含量非常丰富,不易净化。紫甘蓝由于自身基质的复杂性,对其残留的农药进行准确筛查的难度相对较大。本文以可筛查农药种数和假阴性农药种数作为指标,考察了Auto MS/MS和All Ions MS/MS两种不同采集模式,最终选择All Ions MS/MS采集模式。然后在选择的采集模式下,建立了基于LC-QTOF/MS的紫甘蓝中415种农药残留快速筛查方法。将该方法应用于2019年能力验证紫甘蓝中农药残留的筛查和定量,对该方法的准确性和可靠性进行了考察和验证。与传统两次采集进行筛查的方法相比,本文建立的一次进样对紫甘蓝中农药多残留进行定性筛查和定量测定的方法,提高了农药残留筛查的效率,可以为水果和蔬菜中农药多残留的快速筛查提供参考。

## 1 实验部分

### 1.1 仪器、试剂与材料

Agilent 1290 Infinity II LC-6545 Q-TOF/MS液相色谱-质谱联用仪,配有双喷射电喷雾电离源(美国Agilent公司), AH-30全自动均质仪、Fotector Plus高通量全自动固相萃取仪、高通量真空平行浓缩仪(中国睿科仪器有限公司), SR-2DS水平振荡器(日本Tatec公司), KDC-40低速离心机(安徽中佳公司), N-EVAP 112氮吹浓缩仪(美国Organomation Associates公司), Milli-Q超纯水机(美国Millipore公司), PL602-L电子天平(瑞士Mettler-Toledo公司)。

415种农药标准品纯度均≥95%(中国阿尔塔科技有限公司);甲酸和乙酸铵均为质谱纯;乙腈、甲苯为色谱纯(美国Fisher公司);乙酸、氯化钠、无水硫酸钠和无水硫酸镁均为分析纯(中国安谱公司); Carbon/NH_2_柱(美国Agilent公司)。

### 1.2 标准溶液的配制

称取10 mg(精确至0.01 mg)标准品,置于10 mL棕色容量瓶中,以甲醇定容至刻度,配制单标准储备液,于-18 ℃避光保存;根据需要,移取适量储备液,用甲醇稀释,配制所需浓度的标准工作液,于4 ℃避光保存。

### 1.3 样品前处理

提取:称取紫甘蓝样品10 g,置于80 mL离心管中,加入1%醋酸乙腈40 mL,以13500 r/min均质1 min,加入1 g氯化钠、4 g无水硫酸镁,振荡10 min,以4200 r/min离心5 min,取上清液20 mL,在37 ℃、150 r/min条件下蒸发浓缩至约2 mL,待净化。

净化:在Carbon/NH_2_柱中加入约2 cm无水硫酸钠,将Carbon/NH_2_柱放到自动固相萃取仪上。用4 mL乙腈-甲苯(3∶1, v/v)活化SPE柱,并弃去流出液,每次用2 mL乙腈-甲苯(3∶1, v/v)洗涤样液瓶3次,并将洗涤液移入Carbon/NH_2_柱中。再用25 mL乙腈-甲苯(3∶1, v/v)洗脱,合并洗脱液,置于试管中。在37 ℃、150 r/min条件下蒸发浓缩至约0.5 mL,并将浓缩液置于氮气下吹干,加入1 mL乙腈-水(3∶2, v/v)混匀,经0.22 μm滤膜过滤后,定容。

### 1.4 仪器条件

色谱柱:ZORBAX SB-C18柱(100 mm×2.1 mm, 3.5 μm);柱温:40 ℃;流动相:A相为0.1%(v/v)甲酸水溶液(含5 mmol/L乙酸铵), B相为乙腈;流速:0.4 mL/min。梯度洗脱程序:0~3 min, 1%B; 3~6 min, 1%~30%B; 6~9 min, 30%~40%B; 9~15 min, 40%B; 15~19 min, 40%~60%B; 19~23 min, 60%~90%B; 23~23.01 min, 90%~1%B; 23.01~27.01 min, 1%B。进样体积:5 μL。

离子源:双通路喷射流电喷雾电离(Dual AJS ESI)源;扫描方式:正离子全扫描;全扫描范围:*m/z* 50~1000;毛细管电压:4000 V;雾化气体:氮气;雾化气压力:0.14 MPa;鞘气温度:375 ℃;鞘气流速:11.0 L/min;干燥气流速:12.0 L/min;干燥气温度:325 ℃;碎裂电压:145 V。

All Ions MS/MS模式条件:0 min时,碰撞能为0 V; 0.5 min时,碰撞能分别为0、15和35 V。其他质谱参数见表1。

**表1:** 415种农药的分子式、保留时间(*t*_R_)、加合离子、定量离子和定性离子

No.					Compound									Formula						*t*_R_/min							Adduct ion (*m/z*)					Quantitative ion (*m/z*)			Product ion (*m/z*)
1					1,3-diphenyl urea (双苯基脲)									C_13_H_12_N_2_O						7.076							[M+H]^+^	213.1061					94.0642		
2					1-naphthyl acetamide (萘乙酰胺)									C_12_H_11_NO						4.514							[M+H]^+^	186.0912					141.0695		
3					abamectin (阿维菌素)									C_48_H_72_O_14_						18.715							[M+Na]^+^	895.4816					321.9302		
4					acetamiprid (啶虫脒)									C_10_H_11_C_l_N_4_						3.974							[M+H]^+^	223.0745					126.0105		
5					acetochlor (乙草胺)									C_14_H_20_ClNO_2_						12.623							[M+H]^+^	270.1255					133.0886		
6					acibenzolar-*S*-methyl (苯并噻二唑)									C_8_H_6_N_2_OS_2_						9.212							[M+H]^+^	210.9991					55.0552		
7					alachlor (甲草胺)									C_14_H_20_ClNO_2_						12.576							[M+H]^+^	270.1255					238.0993		
8					aldimorph (4-十二烷基-2,6-二甲基吗啉)									C_18_H_37_NO						13.024							[M+H]^+^	284.2948					57.0699		
9					ametoctradin (唑嘧菌胺)									C_15_H_25_N_5_						13.312							[M+H]^+^	276.2183					176.0931		
10					ametryn (莠灭净)									C_9_H_17_N_5_S						6.713							[M+H]^+^	228.1277					186.0808		
11					amidithion (赛硫磷)									C_7_H_16_NO_4_PS_2_						4.342							[M+H]^+^	274.0331					198.9647		
12					aminocarb (灭害威)									C_11_H_16_N_2_O_2_						2.151							[M+H]^+^	209.1285					137.0835		
13					ancymidol (环丙嘧啶醇)									C_15_H_16_N_2_O_2_						5.223							[M+H]^+^	257.1281					81.0435		
14					anilofos (莎稗磷)									C_13_H_19_ClNO_3_PS_2_						14.790							[M+H]^+^	368.0299					198.9631		
15					athidathion (乙基杀扑磷)									C_8_H_15_N_2_O_4_PS_3_						13.354							[M+H]^+^	331.0004					145.0066		
16					atraton (莠去通)									C_9_H_17_N_5_O						4.314							[M+H]^+^	212.1506					68.0243		
17					atrazine (莠去津)									C_8_H_14_ClN_5_						6.441							[M+H]^+^	216.1010					174.0541		
18					azaconazole (氧环唑)									C_12_H_11_C_l2_N_3_O_2_						6.110							[M+H]^+^	300.0301					158.9763		
19					azadirachtin (印楝素)									C_35_H_44_O_16_						6.509							[M+Na]^+^	743.2517					665.2204		
20					azimsulfuron (四唑嘧磺隆)									C_13_H_16_N_10_O_5_S						6.673							[M+H]^+^	425.1099					182.0560		
21					azinphos-ethyl (益棉磷)									C_12_H_16_N_3_O_3_PS_2_						13.380							[M+H]^+^	346.0443					77.0389		
22					azoxystrobin (嘧菌酯)									C_22_H_17_N_3_O_5_						11.168							[M+H]^+^	404.1241					329.0795		
23					beflubutamid (氟丁酰草胺)									C_18_H_17_F_4_NO_2_						14.382							[M+H]^+^	356.1268					91.0542		
24					benalaxyl (苯霜灵)									C_20_H_23_NO_3_						14.105							[M+H]^+^	326.1751					91.0542		
25					benazolin-ethyl (草除灵)									C_11_H_10_ClNO_3_S						10.128							[M+H]^+^	272.0143					169.9821		
26					bendiocarb (口恶虫威)									C_11_H_13_NO_4_						5.827							[M+H]^+^	224.0917					81.0335		
27					benodanil (麦锈灵)									C_13_H_10_INO						8.392							[M+H]^+^	323.9880					230.9301		
28					bensulfuron-methyl (苄嘧磺隆)									C_16_H_18_N_4_O7_S_						7.884							[M+H]^+^	411.0969					149.0597		
29					benthiavalicarb-isopropyl (苯噻菌胺)									C_18_H_24_FN_3_O_3_S						9.387							[M+H]^+^	382.1595					116.0708		
30					benzobicyclon (双环磺草酮)									C_22_H_19_ClO_4_S_2_						13.065							[M+H]^+^	447.0488					229.0316		
31					benzofenap (吡草酮)									C_22_H_20_C_l2_N_2_O_3_						16.231							[M+H]^+^	431.0931					105.0702		
32					benzovindiflupyr (苯并烯氟菌唑)									C_18_H_15_C_l2_F_2_N_3_O						14.427							[M+H]^+^	398.0640					159.0360		
33					bitertanol (联苯三唑醇)									C_20_H_23_N_3_O_2_						12.765							[M+H]^+^	338.1863					70.0400		
34					bixafen (联苯吡菌胺)									C_18_H_12_Cl_2_F_3_N_3_O						13.596							[M+H]^+^	414.0384					394.0317		
35					boscalid (啶酰菌胺)									C_18_H_12_Cl_2_N_2_O						11.302							[M+H]^+^	343.0399					271.0866		
36					bromacil (除草定)									C_9_H_13_BrN_2_O_2_						4.840							[M+H]^+^	261.0233					131.9443		
37					bromfenvinfos-methyl (甲基溴苯烯磷)									C_10_H_10_BrCl_2_O_4_P						11.125							[M+H]^+^	374.8950					127.0155		
38					bromobutide (溴丁酰草胺)									C_15_H_22_BrNO						13.796							[M+H]^+^	312.0959					196.0149		
39					brompyrazon (溴莠敏)									C_10_H_8_BrN_3_O						3.812							[M+H]^+^	265.9924					92.0495		
40					bromuconazole (糠菌唑)									C_13_H_12_BrCl_2_N_3_O						10.459							[M+H]^+^	375.9614					158.9763		
41					bupirimate (乙嘧酚磺酸酯)									C_13_H_24_N_4_O_3_S						12.612							[M+H]^+^	317.1642					44.0495		
No.				Compound				Formula									*t*_R_/min						Adduct ion (*m/z*)						Quantitative ion (*m/z*)					Product ion (*m/z*)	
42				buprofezin (噻嗪酮)				C_16_H_23_N_3_OS									17.415						[M+H]^+^						306.1635					57.0699	
43				butachlor (丁草胺)				C_17_H_26_ClNO_2_									17.522						[M+H]^+^						312.1727					238.0998	
44				butafenacil (氟丙嘧草酯)				C_20_H_18_ClF_3_N_2_O_6_									14.209						[M+NH_4_]^+^						492.1157					331.0092	
45				butamifos (抑草磷)				C_13_H_21_N_2_O_4_PS									16.502						[M+H]^+^						333.1035					180.0600	
46				butocarboxim-sulfoxide (丁酮威亚砜)				C_7_H_14_N_2_O_3_S									2.165						[M+H]^+^						207.0798					46.9950	
47				butoxycarboxim (丁酮砜威)				C_7_H_14_N_2_O_4_S									2.571						[M+H]^+^						223.0747					44.0495	
48				buturon (炔草隆)				C_12_H_13_ClN_2_O									8.039						[M+H]^+^						237.0789					53.0386	
49				cadusafos (硫线磷)				C_10_H_23_O_2_PS_2_									14.780						[M+H]^+^						271.0950					96.9508	
50				cafenstrole (唑草胺)				C_16_H_22_N_4_O_3_S									12.847						[M+H]^+^						351.1491					100.0759	
51				carbaryl (甲萘威)				C_12_H_11_NO_2_									6.291						[M+H]^+^						202.0863					127.0542	
52				carbetamide (双酰草胺)				C_12_H_16_N_2_O_3_									4.663						[M+H]^+^						237.1234					72.0808	
53				carbofuran (克百威)				C_12_H_15_NO_3_									5.869						[M+H]+						222.1125					123.0441	
54				carbofuran-3-hydroxy (三羟基克百威)				C_12_H_15_NO_4_									3.604						[M+H]^+^						238.1074					107.0491	
55				carboxin (萎锈灵)				C_12_H_13_NO_2_S									6.540						[M+H]^+^						236.0740					93.0573	
56				carpropamid (环丙酰菌胺)				C_15_H_18_Cl_3_NO									14.682						[M+H]^+^						334.0524					336.0494	
57				chlorantraniliprole (氯虫苯甲酰胺)				C_18_H_14_BrCl_2_N_5_O_2_									8.358						[M+H]^+^						481.9781					283.9216	
58				chlorbenzuron (灭幼脲)				C_14_H_10_Cl_2_N_2_O_2_									12.952						[M+H]^+^						309.0193					156.0205	
59				chlorbromuron (氯溴隆)				C_9_H_10_BrClN_2_O_2_									9.879						[M+H]^+^						292.9687					203.9205	
60				chlorfenvinphos (毒虫畏)				C_12_H_14_Cl_3_O_4_P									13.776						[M+H]^+^						358.9768					98.9843	
61				chlorimuron-ethyl (氯嘧磺隆)				C_15_H_15_ClN_4_O_6_S									10.766						[M+H]^+^						415.0474					186.0060	
62				chlormequat (矮壮素)				C_5_H_13_ClN									0.750						M^+^						122.0731					58.0657	
63				chlorotoluron (绿麦隆)				C_10_H_13_ClN_2_O									6.149						[M+H]^+^						213.0789					72.0449	
64				chloroxuron (枯草隆)				C_15_H_15_ClN_2_O_2_									10.156						[M+H]^+^						291.0895					72.0449	
65				chlorpyrifos (毒死蜱)				C_9_H_11_Cl_3_NO_3_PS									17.762						[M+H]^+^						349.9336					96.9508	
66				cinosulfuron (醚磺隆)				C_15_H_19_N_5_O_7_S									5.766						[M+H]^+^						414.1078					183.0499	
67				clethodim-sulfoxide (烯草酮亚砜)				C_17_H_26_ClNO_4_S									4.386						[M+H]^+^						376.1344					206.1177	
68				clofentezine (四螨嗪)				C_14_H_8_Cl_2_N_4_									15.403						[M+H]^+^						303.0199					102.0338	
69				clomazone (异口恶草酮)				C_12_H1_4_ClNO_2_									7.997						[M+H]^+^						240.0786					125.0153	
70				clomeprop (氯甲酰草胺)				C_16_H_15_Cl_2_NO_2_									16.600						[M+H]^+^						324.0562					120.0807	
71				cloquintocet-mexyl (解草酯)				C_18_H_22_ClNO_3_									16.812						[M+H]^+^						336.1361					179.0128	
72				clothianidin (噻虫胺)				C_6_H_8_ClN_5_O_2_S									3.538						[M+H]^+^						250.0160					131.9669	
73				coumaphos (蝇毒磷)				C_14_H_16_ClO_5_PS									15.614						[M+H]^+^						363.0217					226.9926	
74				coumaphos-oxon (蝇毒磷-OXON)				C_14_H_16_ClO_6_P									8.411						[M+H]^+^						347.0442					211.0146	
75				coumatetralyl (杀鼠醚)				C_19_H_16_O_3_									11.620						[M+H]^+^						293.1172					91.0542	
76				coumoxystrobin (丁香菌酯)				C_26_H_28_O_6_									17.537						[M+H]^+^						437.1963					145.0649	
77				crimidine (鼠立死)				C_7_H_10_ClN_3_									4.115						[M+H]^+^						172.0636					67.0291	
78				crotoxyphos (巴毒磷)				C_14_H_19_O_6_P									9.667						[M+NH_4_]^+^						332.1259					127.0158	
79				crufomate (育畜磷)				C_12_H_19_ClNO_3_P									10.826						[M+H]^+^						292.0862					108.0192	
80				cumyluron (二苯隆)				C_17_H_19_ClN_2_O									11.013						[M+H]^+^						303.1265					119.0855	
81				cyanazine (氰草津)				C_9_H_13_ClN_6_									5.221						[M+H]^+^						241.0963					214.0854	
82				cyantraniliprole (溴氰虫酰胺)				C_19_H_14_BrClN_6_O_2_									6.976						[M+H]^+^						473.0123					285.9197	
83				cyazofamid (氰霜唑)				C_13_H_13_ClN_4_O_2_S									14.264						[M+H]^+^						325.0521					108.0109	
84				cyclosulfamuron (环丙嘧磺隆)				C_17_H_19_N_5_O_6_S									12.312						[M+H]^+^						422.1130					261.0286	
85				cycloxydim (噻草酮)				C_17_H_27_NO_3_S									16.374						[M+H]^+^						326.1784					107.0491	
86				cycluron (环莠隆)				C_11_H_22_N_2_O									6.487						[M+H]^+^						199.1805					72.0444	
87				cyenopyrafen (腈吡螨酯)				C_24_H_31_N_3_O_2_									18.653						[M+H]^+^						394.2489					254.1285	
88				cymiazole (螨蜱胺)				C_12_H_14_N_2_S									3.840						[M+H]^+^						219.0950					77.0386	
89				cyprazine (环丙津)				C_9_H_14_ClN_5_									6.454						[M+H]^+^						228.1007					186.0535	
90				cyproconazole (环丙唑醇)				C_15_H_18_ClN_3_O									9.316						[M+H]^+^						292.1211					70.0400	
91				cyprodinil (嘧菌环胺)				C_14_H_15_N_3_									11.762						[M+H]^+^						226.1339					93.0573	
92				cyprofuram (酯菌胺)				C_14_H_14_ClNO_3_									6.940						[M+H]^+^						280.0735					69.0340	
93				cyprosulfamide (环丙磺酰胺)				C_18_H_18_N_2_O_5_S									6.040						[M+H]^+^						375.1020					135.0443	
No.					Compound		Formula									*t*_R_/min						Adduct ion (*m/z*)							Quantitative ion (*m/z*)					Product ion (*m/z*)	
94					cyromazine (灭蝇胺)		C_6_H_10_N_6_									0.800						[M+H]^+^							167.1036					68.0246	
95					daimuron (杀草隆)		C_17_H_20_N_2_O									11.259						[M+H]^+^							269.1656					151.0863	
96					demeton-*S*-sulfoxide (内吸磷-*S*-亚砜)		C_8_H_19_O_4_PS_2_									3.602						[M+H]^+^							275.0529					140.9761	
97					desmetryn (敌草净)		C_8_H_15_N_5_S									5.228						[M+H]^+^							214.1121					172.0651	
98					diazinon (二嗪磷)		C_12_H_21_N_2_O_3_PS									15.088						[M+H]^+^							305.1083					96.9508	
99					diclosulam (双氯磺草胺)		C_13_H_10_Cl_2_FN_5_O_3_S									8.244						[M+H]^+^							405.9938					160.9794	
100					dicyclanil (环虫腈)		C_8_H_10_N_6_									1.930						[M+H]^+^							191.1040					92.0243	
101					diethofencarb (乙霉威)		C_14_H_21_NO_4_									9.580						[M+H]^+^							268.1543					124.0393	
102					diethyltoluamide (避蚊胺)		C_12_H_17_NO									6.717						[M+H]^+^							192.1383					91.0542	
103					difenoconazole (苯醚甲环唑)		C_19_H_17_Cl_2_N_3_O_3_									14.631						[M+H]^+^							406.0721					251.0021	
104					difenoxuron (枯莠隆)		C_16_H_18_N_2_O_3_									7.095						[M+H]^+^							287.1390					72.0444	
105					difenzoquat (野燕枯)		C_17_H_17_$N_{2}^{+}$									4.570						[M+H]^+^							249.1386					130.0651	
106					diflufenican (吡氟酰草胺)		C_19_H_11_F_5_N_2_O_2_									16.410						[M+H]^+^							395.0813					266.0412	
107					dimefuron (口恶唑隆)		C_15_H_19_ClN_4_O_3_									8.120						[M+H]^+^							339.1218					72.0449	
108					dimepiperate (哌草丹)		C_15_H_21_NOS									16.076						[M+H]^+^							264.1417					146.0634	
109					dimethachlor (二甲草胺)		C_13_H_18_ClNO_2_									7.736						[M+H]^+^							256.1099					148.1121	
110					dimethametryn (异戊乙净)		C_11_H_21_N_5_S									10.877						[M+H]^+^							256.1590					186.0808	
111					dimethenamid (二甲吩草胺)		C_12_H_18_ClNO_2_S									9.773						[M+H]^+^							276.0820					244.0557	
112					dimethoate (乐果)		C_5_H_12_NO_3_PS_2_									3.831						[M+H]^+^							230.0069					198.9647	
113					dimethomorph (烯酰吗啉)		C_21_H_22_ClNO_4_									8.457						[M+H]^+^							388.1310					165.0546	
114					E-dimethylvinphos (E-甲基毒虫畏)		C_10_H_10_Cl_3_O_4_P									11.580						[M+H]^+^							330.9455					127.0153	
115					Z-dimethylvinphos (Z-甲基毒虫畏)		C_10_H_10_Cl_3_O_4_P									10.593						[M+H]^+^							330.9455					204.9370	
116					dimetilan (敌蝇威)		C_10_H_16_N_4_O_3_									3.858						[M+H]^+^							241.1295					72.0444	
117					dimoxystrobin (醚菌胺)		C_19_H_22_N_2_O_3_									13.187						[M+H]^+^							327.1703					116.0495	
118					diniconazole (烯唑醇)		C_15_H_17_Cl_2_N_3_O									13.054						[M+H]^+^							326.0821					70.0403	
119					dinotefuran (呋虫胺)		C_7_H_14_N_4_O_3_									2.332						[M+H]^+^							203.1139					58.0526	
120					diphenamid (双苯酰草胺)		C_16_H_17_NO									7.950						[M+H]^+^							240.1383					134.0964	
121					dipropyl isocinchomeronate (吡啶酸双丙酯)		C_13_H_17_NO_4_									10.321						[M+H]^+^							252.1240					164.0702	
122					disulfoton sulfone (乙拌磷砜)		C_8_H_19_O_4_PS_3_									8.561						[M+H]^+^							307.0253					96.9512	
123					disulfoton sulfoxide (砜拌磷)		C_8_H_19_O_3_PS_3_									6.378						[M+H]^+^							291.0304					156.9538	
124					ditalimfos (灭菌磷)		C_12_H_14_NO_4_PS									7.500						[M+H]^+^							300.0454					130.0287	
125					dithiopyr (氟硫草定)		C_15_H_16_F_5_NO_2_S_2_									17.197						[M+H]^+^							402.0618					354.0578	
126					diuron (敌草隆)		C_9_H_10_Cl_2_N_2_O									6.721						[M+H]^+^							233.0243					72.0449	
127					edifenphos (敌瘟磷)		C_14_H_15_O_2_PS_2_									13.535						[M+H]^+^							311.0324					109.0107	
128					enestroburin (烯肟菌酯)		C_22_H_22_ClNO_4_									16.986						[M+H]^+^							400.1311					145.0646	
129					epoxiconazole (氟环唑)		C_17_H_13_ClFN_3_O									11.270						[M+H]^+^							330.0806					121.0447	
130					esprocarb (戊草丹)		C_15_H_23_NOS									17.208						[M+H]^+^							266.1575					91.0546	
131					etaconazole (乙环唑)		C_14_H_15_Cl_2_N_3_O_2_									11.068						[M+H]^+^							328.0614					158.9745	
132					ethaboxam (噻唑菌胺)		C_14_H_16_N_4_OS_2_									6.696						[M+H]^+^							321.0838					183.0587	
133					ethiofencarb (乙硫苯威)		C_11_H_15_NO_2_S									6.614						[M+H]^+^							226.0896					107.0491	
134					ethiofencarb-sulfone (乙硫苯威砜)		C_11_H_15_NO_4_S									3.572						[M+NH_4_]^+^							258.0795					107.0491	
135					ethiprole (乙虫腈)		C_13_H_9_Cl_2_F_3_N_4_OS									9.409						[M+H]^+^							396.9899					254.9693	
136					ethirimol (乙嘧酚)		C_11_H_19_N_3_O									3.566						[M+H]^+^							210.1601					98.0600	
137					ethoprophos (灭线磷)		C_8_H_19_O_2_PS_2_									10.958						[M+H]^+^							243.0637					96.9508	
138					etoxazole (乙螨唑)		C_21_H_23_F_2_NO_2_									18.179						[M+H]^+^							360.1770					141.0146	
139					etrimfos (乙嘧硫磷)		C_10_H_17_N_2_O_4_PS									14.606						[M+H]^+^							293.0719					124.9821	
140					famphur (伐灭磷)		C_10_H_16_NO_5_PS_2_									9.454						[M+H]^+^							326.0280					93.0100	
141					fenamidone (咪唑菌酮)		C_17_H_17_N_3_OS									10.942						[M+H]^+^							312.1165					92.0495	
142					fenaminstrobin (烯肟菌胺)		C_21_H_21_Cl_2_N_3_O_3_									16.335						[M+H]^+^							434.1034					170.9763	
143					fenamiphos (苯线磷)		C_13_H_22_NO_3_PS									10.595						[M+H]^+^							304.1131					201.9848	
144					fenamiphos-sulfone (苯线磷砜)		C_13_H_22_NO_5_PS									5.648						[M+H]^+^							336.1029					266.0247	
145					fenamiphos-sulfoxide (苯线磷亚砜)		C_13_H_22_NO_4_PS									4.652						[M+H]^+^							320.1080					108.0573	
No.			Compound								Formula							*t*_R_/min						Adduct ion (*m/z*)					Quantitative ion (*m/z*)					Product ion (*m/z*)	
146			fenarimol (氯苯嘧啶醇)								C_17_H_12_Cl_2_N_2_O							10.688						[M+H]^+^					331.0399					81.0447	
147			fenazaquin (喹螨醚)								C_20_H_22_N_2_O							18.442						[M+H]^+^					307.1805					57.0699	
148			fenbuconazole (腈苯唑)								C_19_H_17_ClN_4_							12.495						[M+H]^+^					337.1219					70.0402	
149			fenfuram (甲呋酰胺)								C_12_H_11_NO_2_							6.755						[M+H]^+^					202.0863					109.0284	
150			fenothiocarb (苯硫威)								C_13_H_19_NO_2_S							12.999						[M+H]^+^					254.1209					72.0441	
151			fenpropidin (苯锈啶)								C_19_H_31_N							7.623						[M+H]^+^					274.2529					147.1168	
152			fenpyrazamine (胺苯吡菌酮)								C_17_H_21_N_3_O_2_S							11.914						[M+H]^+^					332.1427					216.1128	
153			fensulfothion (丰索磷)								C_11_H_17_O_4_PS_2_							7.526						[M+H]^+^					309.0379					140.0290	
154			fensulfothion-oxon (氧丰索磷)								C_11_H_17_O_5_PS							4.071						[M+H]^+^					293.0607					157.0312	
155			fensulfothion-oxon-sulfone (氧丰索磷砜)								C_11_H_17_O_6_PS							4.856						[M+H]^+^					309.0565					252.9932	
156			fenthion-oxon (氧倍硫磷)								C_10_H_15_O_4_PS							7.270						[M+H]^+^					263.0501					44.9793	
157			fenthion-oxon-sulfoxide (氧倍硫磷亚砜)								C_10_H_15_O_5_PS							3.560						[M+H]^+^					279.0451					264.0216	
158			fenthion-sulfoxide (倍硫磷亚砜)								C_10_H_15_O_4_PS_2_							6.057						[M+H]^+^					295.0222					109.0049	
159			fentrazamide (四唑酰草胺)								C_16_H_20_ClN_5_O_2_							15.270						[M+Na]^+^					350.1378					55.0542	
160			flamprop-isopropyl (麦草氟异丙酯)								C_19_H_19_ClFNO_3_							15.141						[M+H]^+^					364.1110					105.0335	
161			flamprop-methyl (麦草氟甲酯)								C_17_H_15_ClFNO_3_							12.283						[M+H]^+^					336.0797					105.0335	
162			flazasulfuron (啶嘧磺隆)								C_13_H_12_F_3_N_5_O_5_S							7.965						[M+H]^+^					408.0584					182.0558	
163			florasulam (双氟磺草胺)								C_12_H_8_F_3_N_5_O_3_S							5.927						[M+H]^+^					360.0373					129.0385	
164			fluacrypyrim (嘧螨酯)								C_20_H_21_F_3_N_2_O_5_							16.712						[M+H]^+^					427.1475					145.0648	
165			fluazifop (吡氟禾草酸)								C_15_H_12_F_3_NO_4_							9.090						[M+H]^+^					328.0791					91.0542	
166			fluazifop-butyl (吡氟禾草灵)								C_19_H_20_F_3_NO_4_							17.726						[M+H]^+^					384.1417					91.0542	
167			fluazuron (啶蜱脲)								C_20_H_10_Cl_2_F_5_N_3_O_3_							17.427						[M+H]^+^					506.0092					141.0135	
168			flubendiamide (氟苯虫酰胺)								C_23_H_22_F_7_IN_2_O_4_S							14.682						[M+H]^+^					705.0125					530.9770	
169			flucycloxuron (氟环脲)								C_25_H_20_ClF_2_N_3_O_3_							17.844						[M+H]^+^					484.1242					132.0437	
170			flufenacet (氟噻草胺)								C_14_H_13_F_4_N_3_O_2_S							13.108						[M+H]^+^					364.0737					124.0557	
171			flufenpyr-ethyl (氟哒嗪草酯)								C_16_H_13_ClF_4_N_2_O_4_							13.922						[M+NH_4_]^+^					409.0573					335.0200	
172			flumetsulam (唑嘧磺草胺)								C_12_H_9_F_2_N_5_O_2_S							4.162						[M+H]^+^					326.0518					129.0385	
173			flumiclorac-Pentyl (氟烯草酸)								C_21_H_23_ClFNO_5_							17.508						[M+H]^+^					441.1584					354.0539	
174			flumorph (氟吗啉)								C_21_H_22_FNO_4_							7.106						[M+H]^+^					372.1606					285.0909	
175			fluometuron (氟草隆)								C_10_H_11_F_3_N_2_O							6.330						[M+H]^+^					233.0896					72.0444	
176			fluopicolide (氟吡菌胺)								C_14_H_8_Cl_3_F_3_N_2_O							11.969						[M+H]^+^					382.9727					172.9556	
177			fluopyram (氟吡菌酰胺)								C_16_H_11_ClF_6_N_2_O							12.235						[M+H]^+^					397.0537					173.0209	
178			fluoroglycofen-ethyl (乙羧氟草醚)								C_18_H_13_ClF_3_NO_7_							17.175						[M+H]^+^					465.0672					343.9925	
179			fluoxastrobin (氟嘧菌酯)								C_21_H_16_ClFN_4_O_5_							13.543						[M+H]^+^					459.0866					188.0380	
180			fluquinconazole (氟喹唑)								C_16_H_8_Cl_2_FN_5_O							11.518						[M+H]^+^					376.0162					349.0043	
181			fluridone (氟啶草酮)								C_19_H_14_F_3_NO							9.350						[M+H]^+^					330.1100					309.0960	
182			flurochloridone (氟咯草酮)								C_12_H_10_Cl_2_F_3_NO							13.147						[M+H]^+^					312.0164					53.0386	
183			flurprimidol (呋嘧醇)								C_15_H_15_F_3_N_2_O_2_							9.461						[M+H]^+^					313.1158					270.0613	
184			flurtamone (呋草酮)								C_18_H_14_F_3_NO_2_							10.082						[M+H]^+^					334.1049					247.0729	
185			flusilazole (氟硅唑)								C_16_H_15_F_2_N_3_Si							12.450						[M+H]^+^					316.1076					165.0697	
186			fluthiacet-methyl (嗪草酸甲酯)								C_15_H_15_ClFN_3_O_3_S_2_							13.855						[M+H]^+^					404.0300					214.9831	
187			flutolanil (氟酰胺)								C_17_H_16_F_3_NO_2_							12.970						[M+H]^+^					324.1206					262.0663	
188			flutriafol (粉唑醇)								C_16_H_13_F_2_N_3_O							6.458						[M+H]^+^					302.1099					70.0400	
189			fluxapyroxad (氟唑菌酰胺)								C_18_H_12_F_5_N_3_O							11.577						[M+H]^+^					382.0973					362.0922	
190			fosthiazate (噻唑磷)								C_9_H_18_NO_3_PS_2_							6.440						[M+H]^+^					284.0538					104.0165	
191			furalaxyl (呋霜灵)								C_17_H_19_NO_4_							9.383						[M+H]^+^					302.1387					95.0128	
192			furametpyr (福拉比)								C_17_H_20_ClN_3_O_2_							6.732						[M+H]^+^					334.1326					157.0163	
193			furathiocarb (呋线威)								C_18_H_26_N_2_O_5_S							17.305						[M+H]^+^					383.1635					195.0474	
194			furmecyclox (拌种胺)								C_14_H_21_NO_3_							13.167						[M+H]^+^					252.1594					55.0542	
195			griseofulvin (灰黄霉素)								C_17_H_17_ClO_6_							7.090						[M+H]^+^					353.0786					69.0335	
196			halosulfuron-methyl (氯吡嘧磺隆)								C_13_H_15_ClN_6_O_7_S							10.124						[M+H]^+^					435.0484					182.0560	
197			haloxyfop-2-ethoxyethyl (氟吡乙禾灵)								C_19_H_19_ClF_3_NO_5_							17.115						[M+H]^+^					434.0977					91.0542	
No.				Compound						Formula							*t*_R_/min						Adduct ion (*m/z*)						Quantitative ion (*m/z*)					Product ion (*m/z*)	
198				haloxyfop-methyl (氟吡甲禾灵)						C_16_H_13_ClF_3_NO_4_							16.301						[M+H]^+^						376.0546					316.0352	
199				hexaconazole (己唑醇)						C_14_H_17_Cl_2_N_3_O							12.285						[M+H]^+^						314.0825					70.0407	
200				hexazinone (环嗪酮)						C_12_H_20_N_4_O_2_							4.727						[M+H]^+^						253.1659					71.0604	
201				imazamethabenz-methyl (咪草酸)						C_16_H_20_N_2_O_3_							4.857						[M+H]^+^						289.1540					144.0437	
202				imazethapyr (咪唑乙烟酸)						C_15_H_19_N_3_O_3_							4.448						[M+H]^+^						290.1499					69.0699	
203				imibenconazole (亚胺唑)						C_17_H_13_C_l3_N_4_S							16.493						[M+H]^+^						410.9999					125.0152	
204				imidacloprid-urea (吡虫啉脲)						C_9_H_10_ClN_3_O							3.303						[M+H]^+^						212.0585					128.0261	
205				imidaclothiz (氯噻啉)						C_7_H_8_ClN_5_O_2_S							3.855						[M+H]^+^						262.0158					181.0537	
206				indaziflam (三嗪茚草胺)						C_16_H_20_FN_5_							9.190						[M+H]^+^						302.1782					145.1007	
207				ipconazole (种菌唑)						C_18_H_24_ClN_3_O							14.230						[M+H]^+^						334.1681					70.0400	
208				ipfencarbazone (三唑酰草胺)						C_18_H_14_Cl_2_F_2_N_4_O_2_							14.896						[M+H]^+^						427.0532					156.0255	
209				iprobenfos (异稻瘟净)						C_13_H_21_O_3_PS							12.403						[M+H]^+^						289.1022					91.0542	
210				iprovalicarb (缬霉威)						C_18_H_28_N_2_O_3_							10.604						[M+H]^+^						321.2173					119.0855	
211				isocarbamid (丁咪酰胺)						C_8_H_15_N_3_O_2_							3.597						[M+H]^+^						186.1237					87.0553	
212				isofenphos-oxon (氧异柳磷)						C_15_H_24_NO_5_P							9.728						[M+H]^+^						330.1465					121.0284	
213				isomethiozin (丁嗪草酮)						C_12_H_20_N_4_OS							13.424						[M+H]^+^						269.1431					57.0699	
214				isoprocarb (异丙威)						C_11_H_15_NO_2_							7.113						[M+H]^+^						194.1176					95.0491	
215				isopropalin (异丙乐灵)						C_15_H_23_N_3_O_4_							18.829						[M+H]^+^						310.1761					188.1308	
216				isoprothiolane (稻瘟灵)						C_12_H_18_O_4_S_2_							12.291						[M+H]^+^						291.0719					144.9776	
217				isoproturon (异丙隆)						C_12_H_18_N_2_O							6.733						[M+H]^+^						207.1492					72.0444	
218				isopyrazam (吡唑萘菌胺)						C_20_H_23_F_2_N_3_O							15.738						[M+H]^+^						360.1895					250.0976	
219				isouron (异口恶隆)						C_10_H_17_N_3_O_2_							5.075						[M+H]^+^						212.1387					72.0451	
220				isoxaben (异口恶酰草胺)						C_18_H_24_N_2_O_4_							12.128						[M+H]^+^						333.1809					165.0546	
221				isoxadifen-ethyl (双苯口恶唑酸)						C_18_H_17_NO_3_							14.478						[M+H]^+^						296.1281					232.0757	
222				isoxathion (口恶唑磷)						C_13_H_16_NO_4_PS							16.343						[M+H]^+^						314.0610					96.9508	
223				kadethrin (噻嗯菊酯)						C_23_H_24_O4S							17.450						[M+H]^+^						397.1468					128.0621	
224				karbutilate (特胺灵)						C_14_H_21_N_3_O_3_							5.606						[M+H]^+^						280.1656					72.0444	
225				lactofen (乳氟禾草灵)						C_19_H_15_ClF_3_NO_7_							17.771						[M+NH_4_]^+^						479.0821					343.9917	
226				linuron (利谷隆)						C_9_H_10_Cl_2_N_2_O_2_							9.223						[M+H]^+^						249.0192					132.9606	
227				malaoxon (马拉氧磷)						C_10_H_19_O_7_PS							5.769						[M+H]^+^						315.0662					99.0077	
228				malathion (马拉硫磷)						C_10_H_19_O_6_PS_2_							12.600						[M+H]^+^						331.0433					99.0077	
229				mandipropamid (双炔酰菌胺)						C_23_H_22_ClNO_4_							11.947						[M+H]^+^						412.1310					125.0153	
230				matrine (苦参碱)						C_15_H_24_N_2_O							0.794						[M+H]^+^						249.1967					148.1117	
231				mecarbam (灭蚜磷)						C_10_H_20_NO_5_PS_2_							13.874						[M+H]^+^						330.0593					142.9385	
232				mefenacet (苯噻酰草胺)						C_16_H_14_N_2_O_2_S							10.954						[M+H]^+^						299.0849					120.0808	
233				mefenpyr-diethyl (吡唑解草酯)						C_16_H_18_Cl_2_N_2_O_4_							15.624						[M+H]^+^						373.0716					159.9715	
234				mefluidide (氟磺酰草胺)						C_11_H_13_F_3_N_2_O_3_S							5.765						[M+H]^+^						311.0672					135.0917	
235				mepanipyrim (嘧菌胺)						C_14_H_13_N_3_							11.591						[M+H]^+^						224.1182					77.0386	
236				mephosfolan (地胺磷)						C_8_H_16_NO_3_PS_2_							4.928						[M+H]^+^						270.0382					139.9566	
237				mepiquat (甲哌)						C_7_H_16_N							0.785						M^+^						114.1283					98.0968	
238				mepronil (灭锈胺)						C_17_H_19_NO_2_							12.270						[M+H]^+^						270.1489					91.0542	
239				mesosulfuron-methyl (甲基二磺隆)						C_17_H_21_N_5_O_9_S_2_							6.811						[M+H]^+^						504.0853					182.0533	
240				metaflumizone (氰氟虫腙)						C_24_H_16_F_6_N_4_O_2_							17.444						[M+H]^+^						507.1250					178.0463	
241				metalaxyl (甲霜灵)						C_15_H_21_NO_4_							6.759						[M+H]^+^						280.1543					45.0335	
242				metamitron (苯嗪草酮)						C_10_H_10_N_4_O							3.527						[M+H]^+^						203.0927					104.0495	
243				metazachlor (吡唑草胺)						C_14_H_16_ClN_3_O							7.546						[M+H]^+^						278.1055					134.0964	
244				metconazole (叶菌唑)						C_17_H_22_ClN_3_O							12.544						[M+H]^+^						320.1524					70.0400	
245				methabenzthiazuron (甲基苯噻隆)						C_10_H_11_N_3_OS							6.008						[M+H]^+^						222.0696					165.0481	
246				methamidophos (甲胺磷)						C_2_H_8_NO_2_PS							0.809						[M+H]^+^						142.0086					63.9947	
247				methidathion (杀扑磷)						C_6_H_11_N_2_O_4_PS_3_							9.202						[M+H]^+^						302.9691					58.0287	
248				methoprotryne (甲氧丙净)						C_11_H_21_N_5_OS							6.563						[M+H]^+^						272.1540					170.0495	
249				methoxyfenozide (甲氧虫酰肼)						C_22_H_28_N_2_O_3_							12.574						[M+H]^+^						369.2173					91.0542	
No.					Compound				Formula								*t*_R_/min						Adduct ion (*m/z*)						Quantitative ion (*m/z*)					Product ion (*m/z*)	
250					metobromuron (溴谷隆)				C_9_H_11_BrN_2_O_2_								7.123						[M+H]^+^						259.0077					91.0416	
251					metolachlor (异丙甲草胺)				C_15_H_22_ClNO_2_								12.407						[M+H]^+^						284.1412					252.1150	
252					metoxuron (甲氧隆)				C_10_H_13_ClN_2_O_2_								4.627						[M+H]^+^						229.0738					72.0444	
253					metrafenone (苯菌酮)				C_19_H_21_BrO_5_								16.315						[M+H]^+^						409.0645					209.0808	
254					monocrotophos (久效磷)				C_7_H_14_NO_5_P								2.809						[M+H]^+^						224.0682					58.0287	
255					monolinuron (绿谷隆)				C_9_H_11_ClN_2_O_2_								6.649						[M+H]^+^						215.0582					98.9996	
256					monuron (灭草隆)				C_9_H_11_ClN_2_O								5.005						[M+H]^+^						199.0633					72.0444	
257					myclobutanil (腈菌唑)				C_15_H_17_ClN_4_								10.672						[M+H]^+^						289.1215					70.0400	
258					naproanilide (萘丙胺)				C_19_H_17_NO_2_								13.581						[M+H]^+^						292.1334					171.0791	
259					napropamide (敌草胺)				C_17_H_21_NO_2_								11.717						[M+H]^+^						272.1645					171.0804	
260					neburon (草不隆)				C_12_H_16_Cl_2_N_2_O								13.219						[M+H]^+^						275.0712					57.0699	
261					norflurazon (氟草敏)				C_12_H_9_ClF_3_N_3_O								7.148						[M+H]^+^						304.0459					140.0306	
262					noruron (草完隆)				C_13_H_22_N_2_O								7.336						[M+H]^+^						223.1811					89.0713	
263					nuarimol (氟苯嘧啶醇)				C_17_H_12_ClFN_2_O								7.391						[M+H]^+^						315.0694					252.0816	
264					ofurace (呋酰胺)				C_14_H_16_ClNO_3_								6.722						[M+H]^+^						282.0891					160.1121	
265					omethoate (氧乐果)				C_5_H_12_NO_4_PS								2.101						[M+H]^+^						214.0297					182.9875	
266					orbencarb (坪草丹)				C_12_H_16_ClNOS								14.900						[M+H]^+^						258.0714					125.0153	
267					orysastrobin (肟醚菌胺)				C_18_H_25_N_5_O_5_								10.332						[M+H]^+^						392.1941					116.0497	
268					oxadixyl (口恶霜灵)				C_14_H_18_N_2_O_4_								5.056						[M+H]^+^						279.1339					132.0808	
269					oxasulfuron (环氧嘧磺隆)				C_17_H_18_N_4_O_6_S								5.326						[M+H]^+^						407.1020					150.0662	
270					oxaziclomefone (口恶嗪草酮)				C_20_H_19_Cl_2_NO_2_								17.367						[M+H]^+^						376.0866					161.0589	
271					oxycarboxin (氧化萎锈灵)				C_12_H_13_NO_4_S								4.462						[M+H]^+^						268.0638					175.0060	
272					oxydemeton-methyl (亚砜磷)				C_6_H_15_O_4_PS_2_								2.656						[M+H]^+^						247.0222					169.0083	
273					paclobutrazol (多效唑)				C_15_H_20_ClN_3_O								8.772						[M+H]^+^						294.1368					70.0400	
274					paraoxon-ethyl (对氧磷)				C_10_H_14_NO_6_P								7.135						[M+H]^+^						276.0632					94.0413	
275					paraoxon-methyl (甲基对氧磷)				C_8_H_10_NO_6_P								5.066						[M+H]^+^						248.0319					109.0049	
276					penconazole (戊菌唑)				C_13_H_15_Cl_2_N_3_								9.981						[M+H]^+^						284.0716					70.0400	
277					pencycuron (戊菌隆)				C_19_H_21_ClN_2_O								15.770						[M+H]^+^						329.1415					125.0153	
278					pendimethalin (二甲戊灵)				C_13_H_19_N_3_O_4_								17.746						[M+H]^+^						282.1448					92.0495	
279					penflufen (氟唑菌苯胺)				C_18_H_24_FN_3_O								13.044						[M+H]^+^						318.1989					141.0460	
280					penoxsulam (五氟磺草胺)				C_16_H_14_F_5_N_5_O_5_S								7.855						[M+H]^+^						484.0709					195.0751	
281					penthiopyrad (吡噻菌胺)				C_16_H_20_F_3_N_3_OS								14.565						[M+H]^+^						360.1362					256.0350	
282					pethoxamid (烯草胺)				C_16_H_22_ClNO_2_								12.450						[M+H]^+^						296.1412					91.0542	
283					phenamacril (氰烯菌酯)				C_12_H_12_N_2_O_2_								5.815						[M+H]^+^						217.0970					104.0497	
284					phorate-oxon-sulfoxide (氧甲拌磷亚砜)				C_7_H_17_O_4_PS_2_								3.497						[M+H]^+^						261.0385					243.0270	
285					phorate-sulfone (甲拌磷砜)				C_7_H_17_O_4_PS_3_								8.647						[M+H]^+^						293.0097					171.0230	
286					phorate-sulfoxide (甲拌磷亚砜)				C_7_H_17_O_3_PS_3_								6.366						[M+H]^+^						277.0150					96.9508	
287					phosalone (伏杀硫磷)				C_12_H_15_ClNO_4_PS_2_								16.038						[M+H]^+^						367.9941					110.9996	
288					phosfolan (硫环磷)				C_7_H_14_NO_3_PS_2_								4.190						[M+H]^+^						256.0217					139.9563	
289					phosmet (亚胺硫磷)				C_11_H_12_NO_4_PS_2_								10.336						[M+H]^+^						318.0018					160.0393	
290					phosmet-oxon (氧亚胺硫磷)				C_11_H_12_NO_5_PS								4.781						[M+H]^+^						302.0252					160.0397	
291					phosphamidon (磷胺)				C_10_H_19_ClNO_5_P								4.730						[M+H]^+^						300.0762					127.0155	
292					benzyl butyl phthalate (邻苯二甲酸丁苄酯)				C_19_H_20_O_4_								16.681						[M+H]^+^						313.1437					91.0547	
293					dicycloethylphthalate (邻苯二甲酸二环己酯)				C_20_H_26_O_4_								18.785						[M+H]^+^						331.1906					149.0229	
294					picaridin (埃卡瑞丁)				C_12_H_23_NO_3_								6.739						[M+H]^+^						230.1751					130.1226	
295					picolinafen (氟吡酰草胺)				C_19_H_12_F_4_N_2_O_2_								17.093						[M+H]^+^						377.0908					238.0474	
296					picoxystrobin (啶氧菌酯)				C_18_H_16_F_3_NO_4_								14.799						[M+H]^+^						368.1104					145.0648	
297					piperalin (哌丙灵)				C_16_H_21_Cl_2_NO_2_								6.045						[M+H]^+^						330.1033					172.9554	
298					piperonyl butoxide (增效醚)				C_19_H_30_O_5_								17.115						[M+NH_4_]^+^						356.2423					177.0898	
299					piperophos (哌草磷)				C_14_H_28_NO_3_PS_2_								16.251						[M+H]^+^						354.1321					142.9385	
300					pirimicarb (抗蚜威)				C_11_H_18_N_4_O_2_								4.424						[M+H]^+^						239.1503					72.0444	
No.	Compound														Formula						*t*_R_/min						Adduct ion (*m/z*)				Quantitative ion (*m/z*)			Product ion (*m/z*)	
301	pirimicarb-desmethyl-formamido														C_11_H_16_N_4_O_3_						5.135						[M+H]^+^				253.1295			72.0444	
	(去甲基-甲酰氨基-抗蚜威)																																		
302	pirimiphos-ethyl (嘧啶磷)														C_13_H_24_N_3_O_3_PS						17.883						[M+H]^+^				334.1349			198.1059	
303	pirimiphos-methyl (甲基嘧啶磷)														C_11_H_20_N_3_O_3_PS						15.909						[M+H]^+^				306.1036			164.1182	
304	pirimiphos-methyl-*N*-desethyl														C_9_H_16_N_3_O_3_PS						7.500						[M+H]^+^				278.0723			67.0297	
	(甲基嘧啶磷-*N*-去乙基)																																		
305	pretilachlor (丙草胺)														C_17_H_26_ClNO_2_						16.252						[M+H]^+^				312.1725			252.1150	
306	primisulfuron-methyl (甲基氟嘧磺隆)														C_15_H_12_F_4_N_4_O_7_S						12.051						[M+H]^+^				469.0436			254.0179	
307	prochloraz (咪鲜胺)														C_15_H_16_Cl_3_N_3_O_2_						13.124						[M+H]^+^				376.0381			70.0287	
308	prodiamine (氨基丙氟灵)														C_13_H_17_F_3_N_4_O_4_						17.238						[M+H]^+^				351.1278			309.0766	
309	profenofos (丙溴磷)														C_11_H_15_BrClO_3_PS						16.193						[M+H]^+^				372.9424			96.9509	
310	prometon (扑灭通)														C_10_H_19_N_5_O						5.303						[M+H]^+^				226.1662			142.0723	
311	prometryn (扑草净)														C_10_H_19_N_5_S						8.679						[M+H]^+^				242.1434			68.0243	
312	propachlor (毒草胺)														C_11_H_14_ClNO						7.458						[M+H]^+^				212.0837			94.0651	
313	propamocarb (霜霉威)														C_9_H_20_N_2_O_2_						2.164						[M+H]^+^				189.1598			74.0237	
314	propaphos (丙虫磷)														C_13_H_21_O_4_PS						13.191						[M+H]^+^				305.0971			44.9793	
315	propaquizafop (口恶草酸)														C_22_H_22_ClN_3_O_5_						16.957						[M+H]^+^				444.1321			56.0495	
316	propiconazole (丙环唑)														C_15_H_17_Cl_2_N_3_O_2_						13.161						[M+H]^+^				342.0771			69.0699	
317	propisochlor (异丙草胺)														C_15_H_22_ClNO_2_						14.387						[M+H]^+^				284.1412			224.0832	
318	propoxur (残杀威)														C_11_H_15_NO_3_						5.734						[M+H]^+^				210.1125			65.0386	
319	propoxycarbazone (丙苯磺隆)														C_15_H_18_N_4_O_7_S						6.080						[M+NH_4_]^+^				399.0951			199.0045	
320	propyzamide (炔苯酰草胺)														C_12_H_11_Cl_2_NO						11.122						[M+H]^+^				256.0290			189.9821	
321	proquinazid (丙氧喹啉)														C_14_H_17_IN_2_O_2_						18.294						[M+H]^+^				373.0407			330.9938	
322	prothioconazole-desthio (脱硫丙硫菌唑)														C_14_H_15_Cl_2_N_3_O						10.546						[M+H]^+^				312.0664			70.0405	
323	prothoate (发硫磷)														C_9_H_20_NO_3_PS_2_						7.874						[M+H]^+^				286.0695			96.9508	
324	pyraclofos (吡唑硫磷)														C_14_H_18_ClN_2_O_3_PS						14.722						[M+H]^+^				361.0537			138.0103	
325	pyraclonil (双唑草腈)														C_15_H_15_ClN_6_						8.320						[M+H]^+^				315.1123			169.0523	
326	pyraclostrobin (吡唑醚菌酯)														C_19_H_18_ClN_3_O_4_						15.474						[M+H]^+^				388.1059			194.0812	
327	pyrametostrobin (唑胺菌酯)														C_21_H_23_N_3_O_4_						12.579						[M+H]^+^				382.1769			163.0621	
328	pyraoxystrobin (唑菌酯)														C_22_H_21_ClN_2_O_4_						14.946						[M+H]^+^				413.1272			145.0649	
329	pyrazolynate (吡唑特)														C_19_H_16_Cl_2_N_2_O_4_S						15.916						[M+H]^+^				439.0281			172.9552	
330	pyrazoxyfen (苄草唑)														C_20_H_16_Cl_2_N_2_O_3_						13.948						[M+H]^+^				403.0612			105.0335	
331	pyridaben (哒螨灵)														C_19_H_25_ClN_2_OS						18.854						[M+H]^+^				365.1449			147.1168	
332	pyridaphenthion (哒嗪硫磷)														C_14_H_17_N_2_O_4_PS						11.685						[M+H]^+^				341.0719			92.0498	
333	pyrifenox (啶斑肟)														C_14_H_12_Cl_2_N_2_O						9.539						[M+H]^+^				295.0391			93.0575	
334	pyrimethanil (嘧霉胺)														C_12_H_13_N_3_						7.556						[M+H]^+^				200.1182			77.0386	
335	pyrimidifen (嘧螨醚)														C_20_H_28_ClN_3_O_2_						16.048						[M+H]^+^				378.1943			184.0631	
336	Z-pyriminobac-methyl (Z-嘧草醚)														C_17_H_19_N_3_O_6_						9.298						[M+H]^+^				362.1338			330.1078	
337	pyrimitate (嘧硫磷)														C_11_H_20_N_3_O_3_PS						14.728						[M+H]^+^				306.1034			170.0748	
338	pyrimorph (丁吡吗啉)														C_22_H_25_ClN_2_O_2_						13.457						[M+H]^+^				385.1677			57.0710	
339	pyriproxyfen (吡丙醚)														C_20_H_19_NO_3_						17.556						[M+H]^+^				322.1438			96.0444	
340	pyroquilon (咯喹酮)														C_11_H_11_NO						4.911						[M+H]^+^				174.0913			117.0573	
341	pyroxsulam (啶磺草胺)														C_14_H_13_F_3_N_6_O_5_S						5.892						[M+H]^+^				435.0693			195.0751	
342	quinoclamine (灭藻醌)														C_10_H_6_ClNO_2_						5.164						[M+H]^+^				208.0160			77.0386	
343	quinoxyfen (喹氧灵)														C_15_H_8_Cl_2_FNO						16.822						[M+H]^+^				308.0040			196.9789	
344	rabenzazole (吡咪唑)														C_12_H_12_N_4_						6.540						[M+H]^+^				213.1135			118.0526	
345	rotenone (鱼藤酮)														C_23_H_22_O_6_						13.259						[M+H]^+^				395.1489			191.0703	
346	saflufenacil (苯嘧磺草胺)														C_17_H_17_ClF_4_N_4_O_5_S						11.031						[M+NH_4_]^+^				501.0617			459.0151	
347	sebuthylazine (另丁津)														C_9_H_16_ClN_5_						7.983						[M+H]^+^				230.1167			174.0541	
348	sebuthylazine-desethyl (去乙基另丁津)														C_7_H_12_ClN_5_						4.580						[M+H]^+^				202.0854			146.0228	
349	secbumeton (仲丁通)														C_10_H_19_N_5_O						5.242						[M+H]^+^				226.1662			170.1036	
No.		Compound											Formula						*t*_R_/min							Adduct ion (*m/z*)				Quantitative ion (*m/z*)				Product ion (*m/z*)	
350		sedaxane (氟唑环菌胺)											C_18_H_19_F_2_N_3_O						11.926							[M+H]^+^				332.1575				159.0361	
351		sethoxydim (烯禾啶)											C_17_H_29_NO_3_S						17.213							[M+H]^+^				328.1941				107.0491	
352		silthiofam (硅噻菌胺)											C_13_H_21_NOSSi						13.434							[M+H]^+^				268.1186				73.0468	
353		simazine (西玛津)											C_7_H_12_ClN_5_						5.039							[M+H]^+^				202.0854				132.0323	
354		simeconazole (硅氟唑)											C_14_H_20_FN_3_OSi						7.355							[M+H]^+^				294.1432				70.0400	
355		simeton (西玛通)											C_8_H_15_N_5_O						3.563							[M+H]^+^				198.1344				100.0509	
356		simetryn (西草净)											C_8_H_15_N_5_S						5.182							[M+H]^+^				214.1121				68.0243	
357		spinetoram (乙基多杀菌素)											C_42_H_69_NO_10_						16.074							[M+H]^+^				748.4994				142.1228	
358		spinosyn A (多杀菌素A)											C_41_H_65_NO_10_						12.820							[M+H]^+^				732.4681				142.1226	
359		spinosyn D (多杀菌素D)											C_42_H_67_NO_10_						14.443							[M+H]^+^				746.4838				142.1226	
360		spiromesifen (螺甲螨酯)											C_23_H_30_O_4_						18.836							[M+H]^+^				371.2217				67.0542	
361		spirotetramat (螺虫乙酯)											C_21_H_27_NO_5_						10.192							[M+H]^+^				374.1962				302.1751	
362		spirotetramat-enol (螺虫乙酯-烯醇)											C_18_H_23_NO_3_						5.330							[M+H]^+^				302.1758				216.1017	
363		spirotetramat-keto-hydroxy (羟基螺虫乙酯酮)											C_18_H_23_NO_4_						5.883							[M+H]^+^				318.1702				268.1333	
364		spirotetramat-mono-hydroxy (单羟基螺虫乙酯)											C_18_H_25_NO_3_						4.673							[M+H]^+^				304.1915				131.0853	
365		sulfentrazone (甲磺草胺)											C_11_H_10_Cl_2_F_2_N_4_O_3_S						6.425							[M+H]^+^				386.9891				306.9944	
366		sulfometuron-methyl (甲嘧磺隆)											C_15_H_16_N_4_O_5_S						5.950							[M+H]^+^				365.0923				150.0660	
367		sulfoxaflor (氟啶虫胺腈)											C_10_H_10_F_3_N_3_OS						4.567							[M+H]^+^				278.0569				174.0517	
368		tebufenpyrad (吡螨胺)											C_18_H_24_ClN_3_O						16.687							[M+H]^+^				334.1681				117.0209	
369		tebupirimfos (丁基嘧啶磷)											C_13_H_23_N_2_O_3_PS						17.615							[M+H]^+^				319.1240				153.1022	
370		tebutam (牧草胺)											C_15_H_23_NO						12.433							[M+H]^+^				234.1852				91.0542	
371		tebuthiuron (丁噻隆)											C_9_H_16_N_4_OS						4.589							[M+H]^+^				229.1118				172.0903	
372		tembotrione (环磺酮)											C_17_H_16_ClF_3_O_6_S						10.078							[M+NH_4_]^+^				458.0646				262.0388	
373		tepraloxydim (吡喃草酮)											C_17_H_24_ClNO_4_						5.459							[M+H]^+^				342.1467				250.1444	
374		terbucarb (特草灵)											C_17_H_27_NO_2_						15.808							[M+H]^+^				278.2116				166.0858	
375		terbufos-oxon (氧特丁硫磷)											C_9_H_21_O_3_PS_2_						10.630							[M+H]^+^				273.0745				57.0704	
376		terbufos-oxon-sulfone (氧特丁硫磷砜)											C_9_H_21_O_5_PS_2_						5.246							[M+H]^+^				305.0635				230.9912	
377		terbufos-oxon-sulfoxide (氧特丁硫磷亚砜)											C_9_H_21_O_4_PS_2_						4.168							[M+H]^+^				289.0700				114.9613	
378		terbufos-sulfone (特丁硫磷砜)											C_9_H_21_O_4_PS_3_						11.802							[M+H]^+^				321.0412				114.9612	
379		terbufos-sulfoxide (特丁硫磷亚砜)											C_9_H_21_O_3_PS_3_						8.401							[M+H]^+^				305.0465				130.9382	
380		terbumeton (特丁通)											C_10_H_19_N_5_O						5.613							[M+H]^+^				226.1662				69.0083	
381		terbuthylazine (特丁津)											C_9_H_16_ClN_5_						8.898							[M+H]^+^				230.1167				174.0541	
382		terbutryn (特丁净)											C_10_H_19_N_5_S						9.090							[M+H]^+^				242.1434				186.0808	
383		tetrachlorvinphos (杀虫威)											C_10_H_9_Cl_4_O_4_P						12.805							[M+H]^+^				364.9065				127.0155	
384		tetraconazole (四氟醚唑)											C_13_H_11_Cl_2_F_4_N_3_O						11.915							[M+H]^+^				372.0290				158.9752	
385		tetramethrin (胺菊酯)											C_19_H_25_NO_4_						17.089							[M+H]^+^				332.1853				286.1806	
386		thenylchlor (噻吩草胺)											C_16_H_18_ClNO_2_S						13.032							[M+H]^+^				324.0819				127.0209	
387		thiacloprid (噻虫啉)											C_10_H_9_ClN_4_S						4.552							[M+H]^+^				253.0309				126.0087	
388		thiazafluron (噻氟隆)											C_6_H_7_F_3_N_4_OS						5.141							[M+H]^+^				241.0365				74.0059	
389		thiazopyr (噻唑烟酸)											C_16_H_17_F_5_N_2_O_2_S						15.484							[M+H]^+^				397.0997				335.0464	
390		thiobencarb (禾草丹)											C_12_H_16_ClNOS						15.229							[M+H]^+^				258.0714				125.0153	
391		tiadinil (噻酰菌胺)											C_11_H_10_ClN_3_OS						10.272							[M+H]^+^				268.0306				101.0170	
392		tiocarbazil (仲草丹)											C_16_H_25_NOS						18.314							[M+H]^+^				280.1724				91.0533	
393		tolfenpyrad (唑虫酰胺)											C_21_H_22_ClN_3_O_2_						16.957							[M+H]^+^				384.1477				197.0956	
394		triadimefon (三唑酮)											C_14_H_16_ClN_3_O_2_						11.262							[M+H]^+^				294.1004				57.0699	
395		triamiphos (威菌磷)											C_12_H_19_N_6_OP						5.538							[M+H]^+^				295.1431				135.0679	
396		triapenthenol (抑芽唑)											C_15_H_25_N_3_O						11.446							[M+H]^+^				264.2066				70.0387	
397		triasulfuron (醚苯磺隆)											C_14_H_16_ClN_5_O_5_S						6.076							[M+H]^+^				402.0633				141.0771	
398		triazophos (三唑磷)											C_12_H_16_N_3_O_3_PS						12.826							[M+H]^+^				314.0723				119.0604	
399		tributyl phosphate (磷酸三丁酯)											C_12_H_27_O_4_P						14.854							[M+H]^+^				267.1710				98.9840	
400		triclopyricarb (氯啶菌酯)											C_15_H_13_Cl_3_N_2_O_4_						16.682							[M+H]^+^				391.0005				210.1487	
401		tricyclazole (三环唑)											C_9_H_7_N_3_S						4.286							[M+H]^+^				190.0433				136.0215	
No.						Compound						Formula						*t*_R_/min							Adduct ion (*m/z*)				Quantitative ion (*m/z*)					Product ion (*m/z*)	
402						trietazine (草达津)						C_9_H_16_ClN_5_						11.432							[M+H]^+^				230.1167					71.0604	
403						trifloxystrobin (肟菌酯)						C_20_H_19_F_3_N_2_O_4_						16.782							[M+H]^+^				409.1370					145.0260	
404						trifloxysulfuron (三氟啶磺隆)						C_14_H_14_F_3_N_5_O_6_S						6.848							[M+H]^+^				438.0690					182.0560	
405						triflumuron (杀铃脲)						C_15_H_10_ClF_3_N_2_O_3_						14.570							[M+H]^+^				359.0405					138.9938	
406						triflusulfuron-methyl (氟胺磺隆)						C_17_H_19_F_3_N_6_O_6_S						12.000							[M+H]^+^				493.1112					264.0698	
407						tri-Isobutyl phosphate (三异丁基磷酸盐)						C_12_H_27_O_4_P						14.537							[M+H]^+^				267.1730					98.9847	
408						trinexapac-ethyl (抗倒酯)						C_13_H_16_O_5_						7.595							[M+H]^+^				253.1071					69.0335	
409						triphenyl phosphate (磷酸三苯酯)						C_18_H_15_O_4_P						14.998							[M+H]^+^				327.0781					77.0386	
410						triticonazole (灭菌唑)						C_17_H_20_ClN_3_O						6.954							[M+H]^+^				318.1368					70.0400	
411						tritosulfuron (三氟甲磺隆)						C_13_H_9_F_6_N_5_O_4_S						9.762							[M+H]^+^				446.0352					145.0258	
412						uniconazole (烯效唑)						C_15_H_18_ClN_3_O						10.667							[M+H]^+^				292.1213					70.0402	
413						valifenalate (霜霉灭)						C_19_H_27_ClN_2_O_5_						10.311							[M+H]^+^				399.1681					116.0741	
414						vamidothion (蚜灭磷)						C_8_H_18_NO_4_PS_2_						3.422							[M+H]^+^				288.0488					58.0287	
415						warfarin (杀鼠灵)						C_19_H_16_O_4_						9.151							[M+H]^+^				309.1121					163.0390	

## 2 结果与讨论

### 2.1 高分辨质谱数据库的建立

在LC-QTOF/MS的一级采集模式下,分别确定每种农药在指定色谱、质谱条件下的保留时间和该农药的离子化形式。大部分农药化合物分子含有较强的电负性元素,如氧原子、氮原子等,在ESI^+^模式下,农药分子中的这些强电负性元素可以和喷雾液滴中的H^+^形成加合离子([M+H]^+^),从而实现检测。样品玻璃瓶和溶剂中会存在微量钠离子,使得一些分子可能与Na^+^也形成较强的加合离子([M+Na]^+^),从而降低了基于[M+H]^+^的检测灵敏度。为此,在流动相的水相中加入适量N $H_{4}^{+}$ ,可以抑制[M+Na]^+^的形成,从而提高检测[M+H]^+^的灵敏度。在所有考察的415种化合物中,有401种化合物可以采用[M+H]^+^检测。另外,有9种化合物需要使用[M+NH_4_]^+^检测,3种化合物使用[M+Na]^+^检测,2种化合物本身在溶液中带正电荷,可以直接检测M^+^。所有415种农药的分子式、加合离子的精确质量数和保留时间等信息,导入MassHunter PCDL Manager软件,形成一级精确质量数据库(PCD库)。在此基础上,在二级采集模式下,分别采集每种农药的母离子在4个不同碰撞能(10、20、30和40 V)下的碎片离子质谱图,并将每个化合物的质谱图导入到上述的PCD库中相应的化合物条目下,从而形成含有二级质谱信息的库—精确质量数据库与谱图库(PCDL)。

### 2.2 采集模式的选择和参数优化

在基于QTOF/MS的高分辨质谱中,要实现同时采集获取一级质谱和二级质谱信息,可以通过两种方式实现。一种是数据依赖的采集模式,如Auto MS/MS采集模式;另外一种是数据非依赖的模式,如All Ions MS/MS采集模式。本文分别对两种模式下的参数进行优化,并在各自最优的条件下进行比较。

2.2.1 Auto MS/MS采集参数的优化

Auto MS/MS采集模式中,可以允许系统根据质谱图中*m/z*的响应强度来自动选择母离子进行二级质谱扫描,同时允许对指定化合物列表优先进行二级质谱扫描。为了保证优化效果,本文根据农药化学性质分类,选择PCDL库中的214种农药放入指定列表,并在其相应的保留时间窗口优先采集其二级质谱图。在固定标准混合溶液质量浓度为100 μg/L条件下,对Auto MS/MS采集模式的参数进行优化。结果显示,在固定一级质谱采集速率下,随着二级质谱采集速率从2 spectra/s增加到6 spectra/s,筛查出的农药占比逐渐下降,同时假阴性农药的占比逐渐增加,采集速率为2 spectra/s时的筛查结果相对最佳,筛查出农药占比可以达到79.91%。因此,在采用Auto MS/MS模式时,二级采集速率建议设定为2 spectra/s,以尽可能提升检出的农药占比,降低假阴性率。

2.2.2 All Ions MS/MS优化

数据非依赖的All Ions MS/MS模式是近年来发展起来的一种高效采集模式,采集时无需事先设置加合离子信息,可以针对采集的数据,依据保留时间、加合离子和二级碎片离子的共流出轮廓匹配情况对化合物进行定性。因此,至少需要高低两个能量通道以满足定性和定量数据的同时采集。碰撞能和采集速率是影响All Ions MS/MS采集模式性能的重要参数,本文分别对二者进行了优化。

实验首先对碰撞能进行了优化。在All Ions MS/MS模式中需要设置碰撞能0 V以获得化合物的加合离子信息,设置0 V以上的碰撞能以获得二级质谱的信息。本实验选择前述214种代表性农药进行碰撞能优化。碰撞能较低时,采集到的碎片离子较少,用于定性的离子可能会偏少,导致定性信息不充分。如果碰撞能较高,化合物的主要定性离子强度降低,同时相对分子量较低的碎片离子增加,而基质中的干扰峰通常位于低相对分子质量端,这会对化合物的定性产生干扰。因此,本文在固定采集速率为2 spectra/s的条件下,对比了在0 V和15 V双碰撞能通道下和0、15和30 V三碰撞能通道下,以及0、15和35 V三碰撞能通道下采集所筛出农药和假阴性农药的占比情况(见图1)。可以看出,在0、15和35 V三碰撞能通道下,可筛查出农药的占比明显高于其他情况,这主要是因为,同时在0、15和35 V三碰撞能通道下采集,可以使得一些相对稳定的化合物产生相对较强的二级碎片离子,有助于对化合物进行同时基于母离子和二级碎片离子的准确定性。

**图1:**
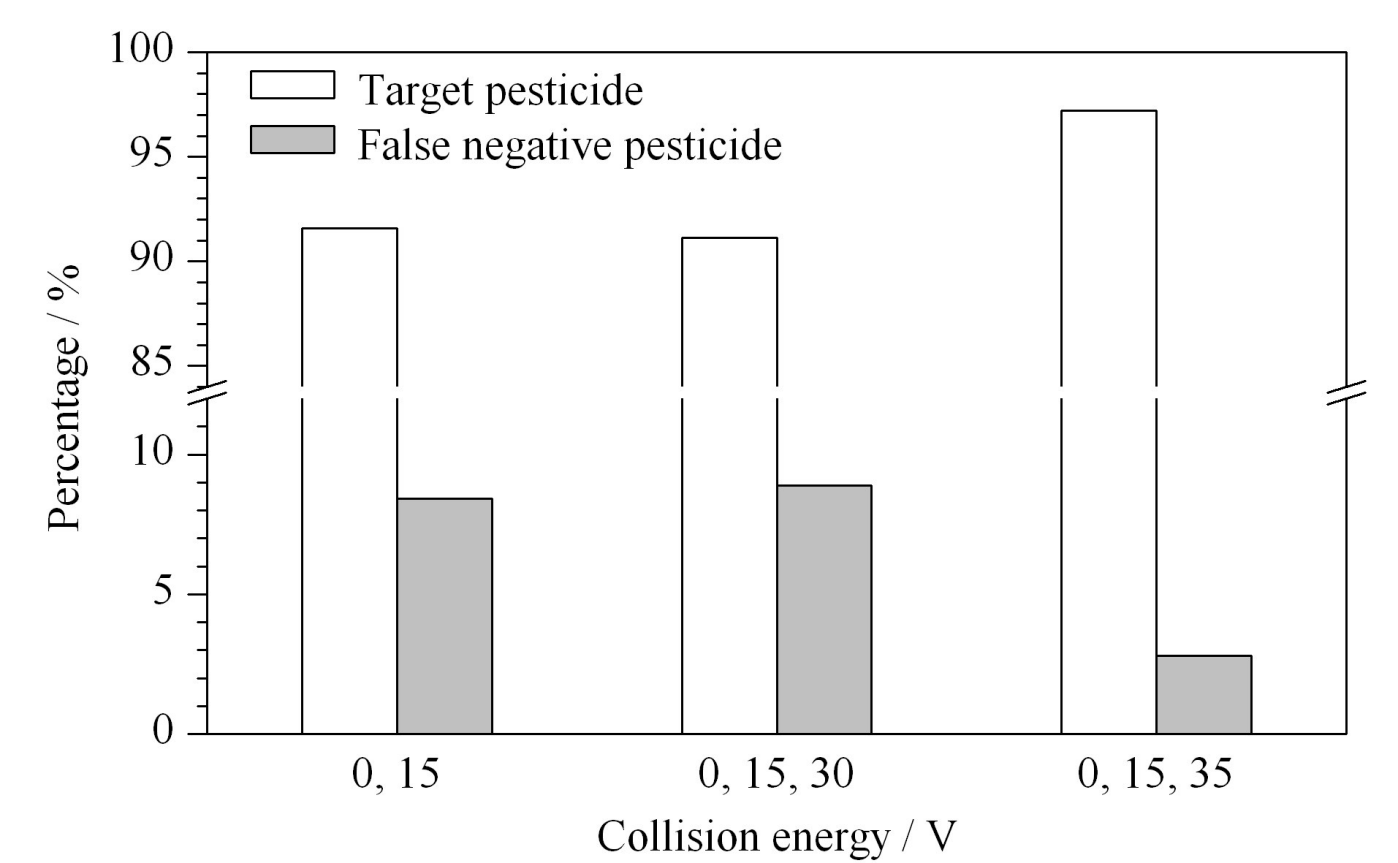
不同碰撞能下准确筛查出的农药和假阴性农药占比

在选定的0、15和35 V三碰撞能通道下,针对这214种农药,对采集速率进行优化。图2给出了2、3、4、5和6 spectra/s等5种采集速率下,筛出农药和假阴性农药的占比情况。从图2可以看出,对于采集速率2、3和4 spectra/s,随着采集速率的增加,可筛查出的农药占比增加,采集速率为4 spectra/s时,所有农药均能筛出,但是当采集速率设置为5 spectra/s和6 spectra/s时,可筛出农药数量的占比有所下降。同时,由于在All Ions MS/MS模式中两个通道交替采集,采集速率逐渐增加,每个采集点的扫描时间下降,使得加合离子的响应逐渐下降,在保证每个色谱峰采集点数满足准确定量的前提下,采集速率为4 spectra/s时的筛查结果最佳。

**图2:**
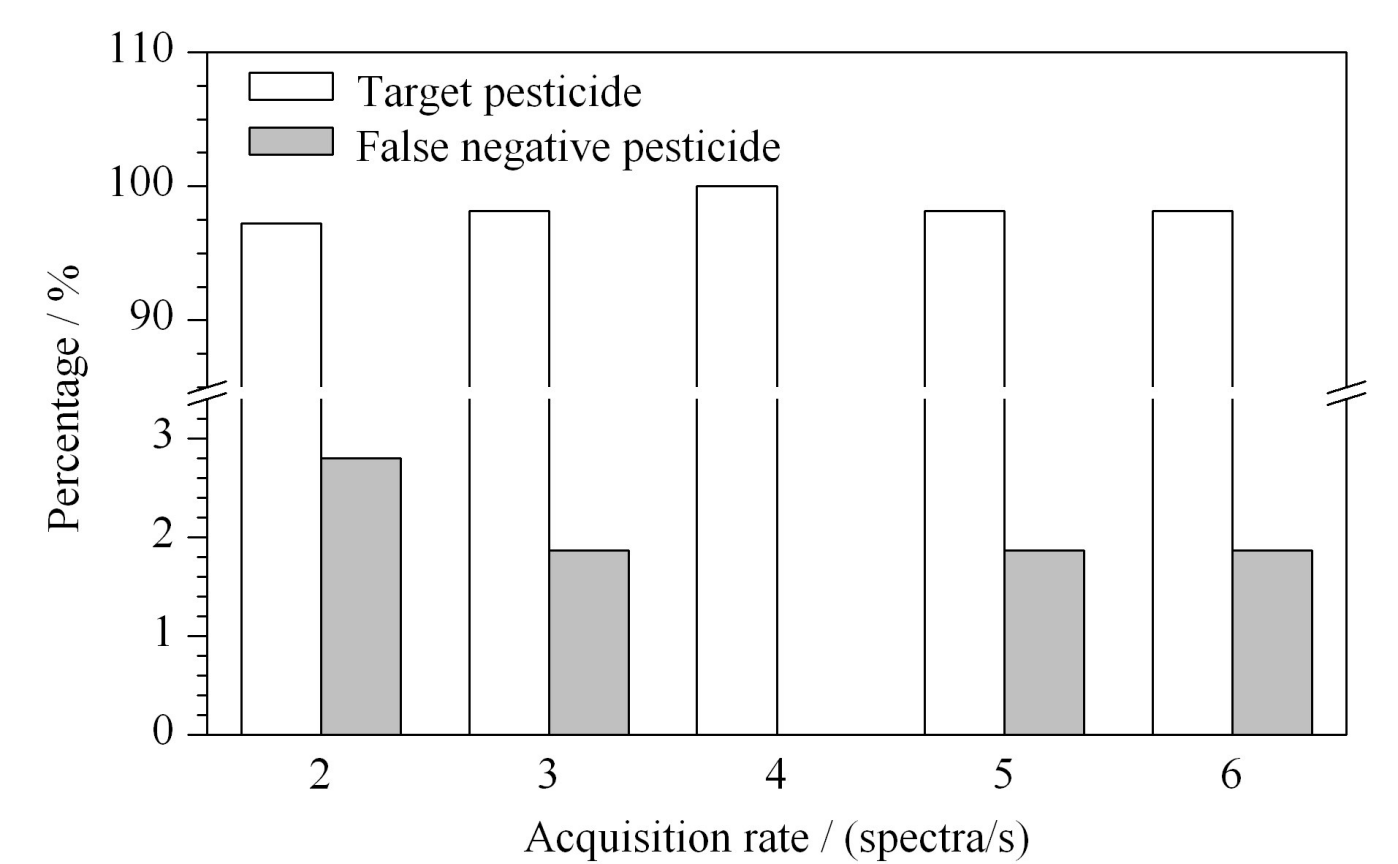
不同采集速率下准确筛查出的农药和假阴性农药占比

2.2.3 不同采集模式的比较

在Auto MS/MS和All Ions MS/MS两种采集模式的最佳条件下,分别对214种农药的筛查情况进行比较。结果表明,214种农药在All Ions MS/MS采集模式下的筛出农药数量占比为100%,远高于在Auto MS/MS采集模式下的检出率(79.91%)。结合前述分析,Auto MS/MS采集模式下的筛出农药占比均低于All Ions MS/MS采集模式任一碰撞能下的检出率,说明All Ions MS/MS采集模式更加灵敏,假阴性率明显降低,优于Auto MS/MS采集模式。因此,本文选择All Ions MS/MS采集模式中0、15和35 V三碰撞能通道和采集速率4 spectra/s作为最佳条件,建立紫甘蓝中415种农药的高通量筛查方法。

### 2.3 方法学验证

2.3.1 线性范围、筛查限与定量限

称取6份空白紫甘蓝样品,按1.3节描述进行前处理后,分别加入不同浓度的混合标准工作液,建立基质匹配标准曲线。应用All Ions MS/MS模式测定目标化合物,以加合离子的质量浓度对其峰面积绘制标准曲线,415种农药在各自范围内线性关系良好,相关系数(*r*^2^)均高于0.990(见附表1,详见http://www.chrom-China.com/)。

关于筛查限(SDL)的确定,参照欧盟指南文件SANTE/12682/2019的要求,在一系列浓度水平上做添加回收试验,每个水平制备20个平行样,按照1.3节描述进行前处理后进行检测。如果在某添加水平下,某个化合物在19个紫甘蓝样品中均能被定性筛查出来,则该浓度被确定为该化合物在紫甘蓝中的筛查限。附表1给出了415种农药的筛查限,为1~20 μg/kg,其中411种农药的筛查限小于等于5 μg/kg。

按照1.3节描述对紫甘蓝样品进行前处理,向空白样品中添加不同水平的目标化合物,在前述最佳条件下进行测定,以信噪比*S/N*≥10对应的添加水平作为定量限(LOQ),结果见附表1,紫甘蓝中415种农药的定量限为1~20 μg/kg,其中413种农药的LOQ≤10 μg/kg。

2.3.2 回收率与精密度

为了考察方法的准确度和精密度,对空白紫甘蓝样品进行添加回收试验。分别选择1倍、2倍和10倍LOQ的混合标准工作液作为添加水平,按前述方法进行前处理,每个添加水平制备5个平行样品。同时进行空白实验,扣除本底值后计算添加回收率和相对标准偏差。紫甘蓝中415种农药在1倍、2倍和10倍LOQ添加水平下的回收率分别为65.7%~118.4%、72.0%~118.8%和70.2%~111.2%,相对标准偏差分别为0.9%~19.7%、0.2%~19.9%和0.6%~19.9%,结果见附表1。说明该方法的准确度和精密度满足准确定量的要求。

### 2.4 欧盟能力验证样品测定

2019年欧盟组织了紫甘蓝中农药残留检测的能力验证,包括定性筛查(EUPT-SM-11)和定量筛查(EUPT-FV-21)两方面内容。将本文建立的筛查方法应用于该能力验证项目提供的紫甘蓝样品中农药残留的定性筛查和准确定量,评估了该方法的定性可靠性和定量准确性。样品前处理按照1.3节描述进行,用All Ions MS/MS模式在最佳参数条件下进行数据采集。

定性筛查EUPT-SM-11未给定紫甘蓝样品中添加农药的范围,重点考察筛查出农药的种类,定量仅做参考,而且要求收到样品72 h内上报结果。结果显示,本实验室应用本文开发的方法筛出了添加的16种农药,没有假阳性结果。与欧盟官方最终公布结果完全一致。

关于定量筛查EUPT-FV-21,组织方提供了强制和自愿农药清单,要求首先对紫甘蓝中在清单范围内的农药进行筛查,再对筛查出的农药准确定量。要求检测范围覆盖强制农药清单的90%,检出所添加农药种类的90%或以上并能准确定量,并且不能出现假阳性结果。

应用本文建立的方法首先进行定性筛查,准确筛查出了其中可以采用基于液相色谱-质谱技术检测的19种非挥发性农药,并采用相应的标准品进行进一步的准确定量。结果表明,本文建立的紫甘蓝中农药多残留筛查方法定性能力强,没有假阳性,而且定量结果准确可靠,所有筛查出的化合物的定量结果均在验证样品的标准值允许误差范围内。

## 3 结论

通过对比不同采集模式,优化采集参数,本文应用液相色谱-四极杆-飞行时间质谱建立了紫甘蓝中415种农药残留的高通量定性筛查与准确定量方法。将该方法应用于2019年欧盟组织的紫甘蓝中农药残留能力验证,结果表明,基于All Ions MS/MS采集模式的一次进样便可快速定性和定量的方法,具有非常强的定性筛查能力,同时可以对筛查出的化合物进行准确定量。该方法具有简单、快速的特点,适用于对紫甘蓝中多种农药残留的快速筛查,可以为其他水果蔬菜中农药残留的高通量筛查提供参考。

## References

[1] Pang G F, Fan C L, Chang Q Y, et al. Food Science, 2012,33(S1):1

[2] Shendy A H, Al-Ghobashy M A, Mohammed M N, et al. J Chromatogr A, 2015,1427(1):142 26687165 10.1016/j.chroma.2015.11.068

[3] Garcia M D, Duque S, Fernández, A B, et al. Talanta, 2017. 163(2):54 27886770 10.1016/j.talanta.2016.10.083

[4] Sapozhnikova Y, Lehotay S J. J Agric Food Chem, 2015,63(21):5163 25686151 10.1021/jf506256q

[5] Zeying H, Lu W, Yi P, et al. Food Chem, 2015,169(4):372 25236240 10.1016/j.foodchem.2014.07.102

[6] Chang Q Y, Pang G F, Fan C L, et al. J AOAC Int, 2016,99(4):1049 27151741 10.5740/jaoacint.16-0063

[7] Chen Y, Lopez S, Hayward D G, et al. J Agric Food Chem, 2016,64(31):6125 27101866 10.1021/acs.jafc.6b00746

[8] Dias Jonatan V, Cutillas V, Lozano A, et al. J Chromatogr A, 2016,1462(1):8 27507727 10.1016/j.chroma.2016.07.072

[9] Wang J, Cheung W. J AOAC Int, 2016,99(2):539 26965037 10.5740/jaoacint.15-0265

[10] Hanot V, Goscinny S, Deridder M, et al. J Chromatogr A, 2015,1384(1):53 25660522 10.1016/j.chroma.2015.01.040

[11] Zhao M A, Feng Y N, Zhu Y Z, et al. J Agric Food Chem, 2014,62(47):11449 25380470 10.1021/jf504570b

[12] Morris B D, Schriner R B. J Agric Food Chem, 2015,63(21):5107 25702899 10.1021/jf505539e

[13] Jadhav M R, Oulkar D P, Ahammed S T P, et al. J Agric Food Chem, 2015,63(18):4449 25639652 10.1021/jf505221e

[14] Gomezperez M L, Romerogonzalez R, Martinez V J, et al. Talanta, 2015,131(1):1 25281065

[15] Liesner M. Chem Unserer Zeit, 2003,37(2):155

[16] Wang Z B, Chang Q Y, Kang J, et al. Anal Methods, 2015,7(15):6385

[17] Wang Z B, Cao Y Z, Ge N, et al. Anal Bioanall Chem, 2016,408(27):7795 10.1007/s00216-016-9883-3 27558104

[18] Botitsi H V, Garbis S D, Economou A, et al. Mass Spectrom Rev, 2011,30(5):907 24737632 10.1002/mas.20307

[19] Gómez-Ramos M M, Ferrer C, Malato O, et al. J Chromatogr A, 2013,1287(1):24 23535633 10.1016/j.chroma.2013.02.065

[20] Zhao Z Y, Shi Z H, Kang J, et al. Chinese Journal of Chromatography, 2013,31(4):372 23898638 10.3724/sp.j.1123.2013.01034

[21] Yu L, Song W, Lü Y N, et al. Chinese Journal of Chromatography, 2015,33(6):597 26536763 10.3724/sp.j.1123.2015.02056

[22] Kang J, Fan C L, Cao Y F, et al. Anal Methods, 2014,6(20):8337

[23] Broecker S, Herre S, Bernhard W, et al. Anal Bioanal Chem, 2011,400(1):101 21127842 10.1007/s00216-010-4450-9

[24] Zhou X J, Chen Y, Yang S J, et al. Chinese Journal of Chromatography, 2017,35(8):787 29048811 10.3724/SP.J.1123.2017.03024

[25] Liu Y Q, Liu S, Xu W J, et al. Chinese Journal of Chromatography, 2017,35(9):941 29048851 10.3724/SP.J.1123.2017.06001

